# Chronic Heat Stress Inhibits Immune Responses to H5N1 Vaccination through Regulating CD4^**+**^CD25^**+**^Foxp3^**+**^ Tregs

**DOI:** 10.1155/2013/160859

**Published:** 2013-09-17

**Authors:** Di Meng, Yanxin Hu, Chong Xiao, Tangting Wei, Qiang Zou, Ming Wang

**Affiliations:** ^1^Key Laboratory of Zoonosis of Ministry of Agriculture, College of Veterinary Medicine, China Agricultural University, No. 2 Yuanmingyuan, West Road, Beijing 100193, China; ^2^Institute of Laboratory Animal Science, Chinese Academy of Medical Science, Beijing 100021, China; ^3^State Key Laboratory for Agrobiotechnology, China Agricultural University, Beijing 100193, China; ^4^Zhongmu Institutes of China Animal Husbandry Group, No. 156 Beiqing Road, Haidian District, Beijing 100095, China

## Abstract

Chronic heat stress (CHS) is known to have negative impacts on the immune responses in animals and increases their susceptibility to infections including the highly pathogenic avian influenza virus H5N1. However, the role of regulatory T cells (Tregs) in CHS immunosuppression remains largely undefined. In this study, we demonstrated a novel mechanism by which CHS suppressed both Th1 and Th2 immune responses and dramatically decreased the protective efficacy of the formalin-inactivated H5N1 vaccine against H5N1 influenza virus infection. This suppression was found to be associated with the induced generation of CD4^+^CD25^+^FoxP3^+^ Tregs and the increased secretions of IL-10 and TGF-**β** in CD4^+^ T cells. Adoptive transfer of the induced Tregs also suppressed the protective efficacy of formalin-inactivated H5N1 virus immunization. Collectively, this study identifies a novel mechanism of CHS immunosuppression mediated by regulating CD4^+^CD25^+^Foxp3^+^ Tregs.

## 1. Introduction

Chronic heat stress (CHS) occurs under the high temperature conditions which are not necessarily extreme but for a long time, for example, in the hot summer. Although prolonged exposure to these environments may not be lethal, it can alter the animal's growth performance, the immune competence, and disease resistance [1–7]. Our previous studies had demonstrated that CHS significantly inhibited both systemic and local innate immune responses, including reduced numbers of pulmonary alveolar macrophages (PAMs), delayed maturation of dendritic cells (DCs), and decreased levels of IL-6, IFN-*β*, and HSP70 mRNA [2]. In addition, CHS caused respiratory system lesions and increased the susceptibility of animals to the highly pathogenic avian influenza virus (HPAIV) H5N1 [2]. Moreover, CHS reduced adaptive immunity to DNA vaccination by mainly suppressing Th1 immune responses, including IgG2a production, T-cell proliferation, IFN-*γ* production by CD4^+^ and CD8^+^ T cells, and antigen specific CTL activities *in vivo* [1]. In short, CHS that can suppress both the innate and adaptive immune responses have been explored. However, the role of regulatory T cells (Tregs)—a subset of T cells critical for immunosuppression—remained to be defined. 

In the recent years, a functionally unique subset of T cells known as CD4^+^CD25^+^ Tregs was identified, which conversed from CD4^+^CD25^−^ Tregs in the periphery after being stimulated by T-cell receptor and TGF-*β* [8, 9]. CD4^+^CD25^+^ Tregs engaged in the maintenance of immunological self-tolerance and downregulated various immune responses through inhibiting the activation of the targeted effector cells in a cell-cell contact dependent manner or indirectly through the secretions of suppressive factors such as IL-10 and TGF-*β* [10, 11]. These Tregs express high levels of the cell surface molecules CD25, GITR, and CTLA-4 and are characterized by the expression of a member of the forkhead/winged-helix transcription factors, FOXP3, which serves as a master regulator gene for the development and function of these cells [12–15]. 

In the present study, we demonstrated a novel mechanism of CHS immunosuppression: both Th1 and Th2 responses were reduced through the regulating CD4^+^CD25^+^FoxP3^+^ Tregs and the secretions of IL-10 and TGF-*β*, demonstrating that Tregs actively participate in the reduced adaptive immune response following CHS.

## 2. Materials and Methods

### 2.1. Ethics Statement

All animal protocols were strictly complied with the guidelines of Beijing Laboratory Animal welfare and Ethical Guidelines of Beijing Administration Committee of Laboratory Animals and were approved by the Beijing Association for Science and Technology, the approve ID is SYXK (Beijing) 2010-0823.

### 2.2. Virus

The H5N1 influenza virus (A/Chicken/Henan/1/04) used in this study was the same as previously described [2]. The virus was propagated in the allantoic cavities of 10-day-old chicken egg embryos for 48 h at 37°C. After incubation, the allantoic fluid was harvested, aliquoted, and stored at −80°C. Viral titers were determined by plaque assays, and the LD_50_ was determined in mice as previously described [16].

### 2.3. Chronic Heat Stress Model

**Figure 1:**
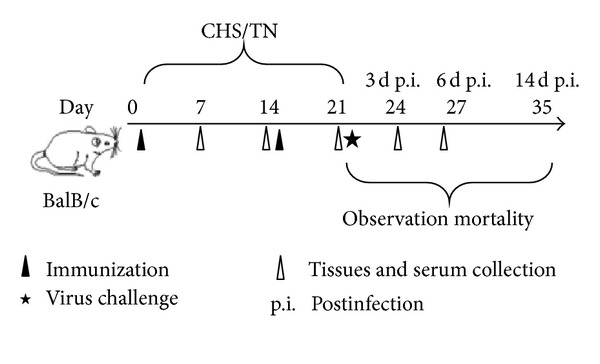
Experimental design schematic. Mice were randomly divided into four groups. Mice under chronic heat stress (CHS) were exposed to 38 ± 1°C for 4 h per day from day 0 to day 21, meanwhile mice of thermally neutral (TN) groups were maintained at 24 ± 1°C. Mice were immunized with 50 *μ*L formalin-inactivated H5N1 virus or PBS on day 0 and boosted on day 14, and all the mice were challenged with PBS-diluted H5N1 virus (50 PFU) on day 21. The mortality of virus-infected mice was observed for 14 days after the viral challenge. Three mice from each group were sacrificed on days 7, 14, 21, 24, and 27; serum, lung, and spleen tissues were collected.

Female BALB/c mice at 8–10 weeks old were purchased from Vital River Laboratories (Beijing, China) and were fed with pathogen-free food and water in independent ventilated cages. Mice were randomly divided into a chronic heat stress (CHS) group and a thermally neutral (TN) group (*n* = 60) (see Figure 1). Mice in CHS groups were treated at 38 ± 1°C in a biological oxygen demand (BOD) incubator for 4 h every day, meanwhile mice in the TN groups were kept at 24 ± 1°C. Both of the incubations were conducted for 21 consecutive days.

### 2.4. Inactivation of Virus and Immunization

In order to inactiate the virus, formalin was added to the virus solution to a final concentration of 0.2%, and the inactivation was carried out for 24 h at 37°C. The immunogenicity of this inactivated virus was tested in mice, and the virus was unable to infect chicken embryos. Half of mice in CHS and TN groups were immunized subcutaneously on day 0 and boosted on day 14 with 50 *μ*L of formalin-inactivated virus, and the remaining mice received 50 *μ*L phosphate-buffered saline (PBS) as a control. Serum were collected on days 7, 14, and 21 (three mice per group each time) after the primary immunization and stored at −20°C.

### 2.5. Viral Challenge

Mice were anesthetized with Zotile (Virbac, France) and intranasally infected with PBS-diluted H5N1 virus (50 PFU) on day 7 after the secondary immunization. These mice were observed for 14 days after the viral challenge. Three mice each group were sacrificed on day 3 and day 6 postinfection, respectively, and lung and spleen tissues were collected and stored in liquid nitrogen until required.

### 2.6. Quantitative Real-Time PCR

About 10 mg lung or spleen tissues were homogenized in Trizol reagents (Invitrogen, Carlsbad, USA), total RNA was extracted, and cDNA was reversely transcribed using an EasyScript First-Strand cDNA Synthesis Super Mix (TransGen Biotech, China) according to the manufacturer's instruction. Real-time PCR reaction was performed in triplicate using a Power SYBR Green PCR Master Mix kit (Applied Biosystems, Warrington, UK), the reaction was run on an Applied Biosystems 7500 system. The copy number of the H5N1 virus hemagglutinin (HA) gene was measured using the absolute quantification method as previously described [2], the following primers were used: forward primer, 5′-CGC AGT ATT CAG AAG AAG CAA GAC-3′, and reverse primer, 5′-TCC ATA AGG ATA GAC CAG CTA CCA-3′.

Single splenocyte suspensions were prepared, and CD4^+^ T cells were isolated and purified using the CD4^+^ T-Cell Isolation Kit II according to the manufacturer's protocol (Miltenyi Biotec, Order no. 130-095-248, USA). Purity of each cell preparation was 95%. 10^6^ CD4^+^ T cells were obtained for real-time PCR.

The expression level of TGF-*β* (forward primer, 5′-GCA ACA TGT GGA ACT CTA CCA GAA-3′, reverse primer, 5′-GAC GTC AAA AGA CAG CCA CTC A-3′) and IL-35 (forward primer, 5′-CTTTGTGGCTGAGCGAAT-3′, reverse primer, 5′-CAGTCACTTGGTTTCCCATA-3′) was conducted using the relative quantification method, which was normalized to the result of the control groups using the 2-ΔΔCT method with *β*-actin (forward primer, 5′-GAG ACC TTC AAC ACC CCG C-3′, reverse primer, 5′-ATG TCA CGC ACG ATT TCC C-3′) as an internal standard. The amplifications were performed as follows: 10 min at 95°C, followed by 40 cycles of 95°C for 15 s, 55°C for 30 s, and 72°C for 40 s.

### 2.7. Detection of Anti-H5N1-HPIV Antibody

Anti-H5N1 antibody levels in the serum were assayed by enzyme-linked immunosorbent assay (ELISA). Ninety-six-well polystyrene microtiter plates (Costar, USA) were coated with inactivated virus (2 *μ*g/mL per well) overnight at 4°C. After blocking, plates were incubated with the mice serum samples in a serial dilution at 37°C for 1 h. Then, a 1 : 2000 dilution HRP-conjugated goat anti-mouse IgG, IgG1, and IgG2a (Proteintech, USA) were added. After one hour incubation, the tetramethylbenzidine substrate (BD Biosciences, USA) was added, and the reaction stopped by 0.2 M H_2_SO_4_. OD values at 450/620 nm were measured with a microplate reader (iMark, Biorad, USA). The serum titer values of total IgG, IgG1, and IgG2a isotypes were assigned as the highest dilution that gave an above 2.1 ratio between testing serum and the untreated negative control. 

### 2.8. Haemaglutination Inhibition (HI) Test

HI test was carried out to detect influenza virus antibodies in serum samples as described elsewhere (World Health Organization, 2002). Briefly, 4 hemagglutinating units (HAU) of virus were prepared according to the HA titers, then serum samples with a twofold dilution were mixed 1 : 1 with 25 *μ*L 4 HAU virus; the mixture was incubated at room temperature for 15 min, then 50 *μ*L erythrocytes were added and incubated at room temperature for the appropriate time. The HI titer was the reciprocal of the last dilution of the serum that completely inhibits haemaglutination.

### 2.9. T-Cell Proliferation

Single lymphocyte suspensions were obtained from spleens of mice in each group (*n* = 3) on day 7 after the second immunization (day 21). Cells in RPMI-1640 containing 10% fetal bovine serum (FBS) were stimulated *in vitro* for 48 h with concanavalin A (ConA, positive control), 20 *μ*g/mL of antigen pool from inactivated H5N1 virus (specific antigen), 2 *μ*g/mL of bovine serum albumin (BSA, irrelevant antigen) or no antigen (negative control), respectively. Following stimulation, a 20 *μ*L mixture of MTS/PMS (Promega, USA) reagent was added and incubated for 4 h, the samples were centrifuged at 500 ×g for 5 min, and then 100 *μ*L DMSO was added (AMRESCO, USA). The OD values of plates were determined at 490 nm using a plate reader (Magellan, Tecan Austria GmbH). Data were shown as the stimulation index (SI), which is calculated as the mean reading of triplicate antigen stimulated wells, divided by the mean reading of triplicate control wells.

### 2.10. Flow Cytometric (FACS) Analysis

Three mice from each group were sacrificed during CHS, and single cell suspensions were isolated from spleens. Cells were fixed with 4% paraformaldehyde and permeabilized with 0.1% saponin (Sigma, Aldrich) to perform the intracellular staining. Cells were then stained with the antibodies for 30 min at 4°C. All antibodies used in this study were purchased from eBiosciences (San Diego, USA), including PE-conjugated anti-CD4, APC-conjugated anti-CD8, FITC-conjugated anti-IFN-*γ*, APC-conjugated anti-CD4, CY5-conjugated anti-IL-4, FITC-conjugated anti-CD4, PE-conjugated anti-IL-10, CY5-conjugated anti-CD25, and APC-conjugated anti-Foxp3. A FACS Calibur was used to measure fluorescent intensities, and the data were analyzed using the Cell Questpro software (BD Biosciences, USA). Splenic T cells were stimulated with a viral-specific antigen for 6 h *in vitro* before intracellular staining of IL-10^+^ CD4^+^ T cells. 

### 2.11. Isolation of Tregs and Adoptive Transfer

Single splenocyte suspensions of CHS and TN immunized mice were prepared. CD4^+^CD25^+^ Tregs were isolated and purified using the MagCellect Mouse CD4^+^CD25^+^ T Cell Isolation Kit according to the manufacturer's protocol (R&D Systems, Inc., Minneapolis, USA). Purity of each cell preparation was 90–95%. 10^6^ cells were adoptively transferred intravenously into TN immunized mice. 

### 2.12. Histopathological Analysis

Tissues were fixed with 4% neutral formalin for 48 h at room temperature, embedded in paraffin, and then cut to 5 *μ*m thickness. Each slide was stained with hematoxylin and eosin (H&E) then examined by light microscopy (Olympus BX41). 

### 2.13. Statistical Analysis

Differences between two groups were determined by using Student's *t* test. A one-way ANOVA test was used for multiple group comparisons. The difference between two groups was considered significant when the *P* value was ≤0.05.

## 3. Results

### 3.1. The Protective Efficacy of Formalin-Inactivated H5N1 Virus Immunization Is Weakened during CHS

**Figure 2:**
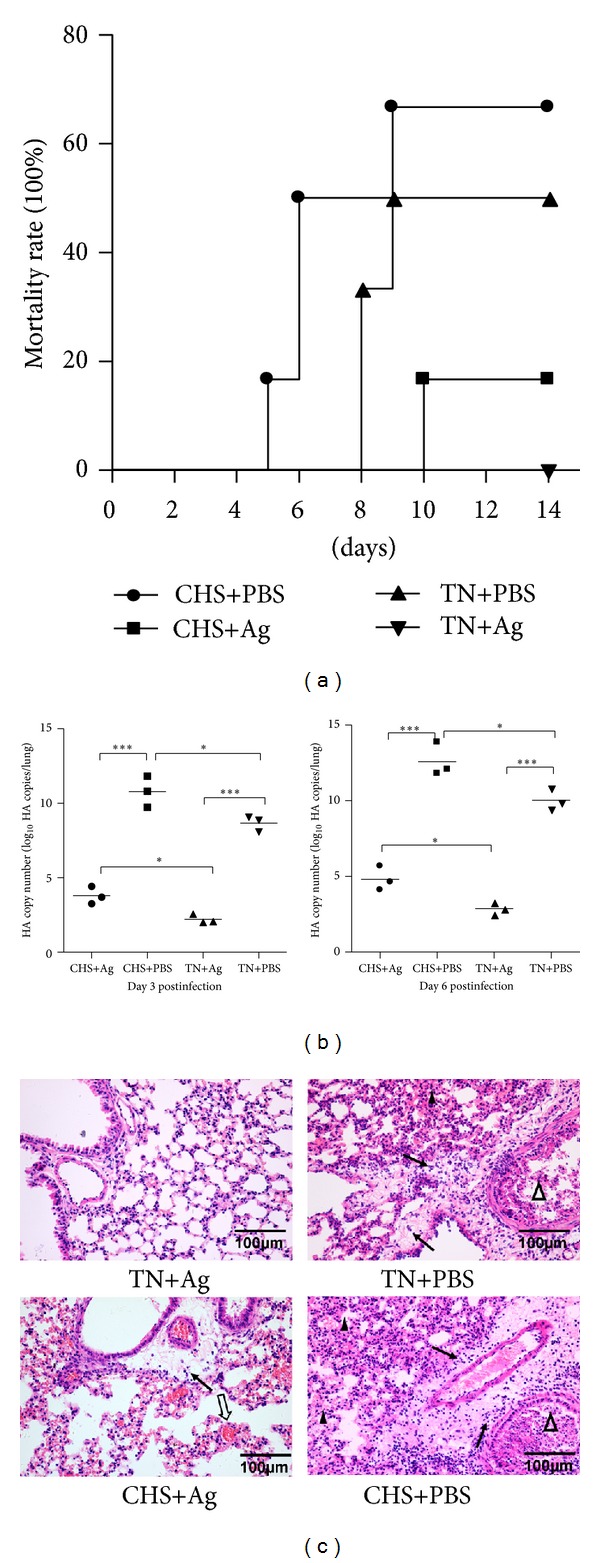
The protective efficacy of formalin-inactivated H5N1 virus immunization is weakened during CHS. After CHS/TN and immunization, all the mice were challenged with PBS-diluted H5N1 virus (50 PFU). (a) The mortality of each group (*n* = 6 per group) was observed for 14 days after viral challenge. (b) Viral loads in the lungs at indicated days postinfection (*n* = 3 per group) were estimated by real-time PCR. Shown are representative results from three independent experiments. Statistically significant differences between the control and treated groups are indicated by ***(*P* < 0.001) and *(*P* < 0.05), respectively. (c) Representative lung sections from each group were stained with H&E. Solid arrows indicate interstitial oedema and inflammatory cellular infiltration about blood vessels, open triangles indicate dropout of mucous epitheliums and inflammatory cellular infiltration in bronchioles, open arrows indicate congestion, and solid triangles indicate thickening of the alveolar walls (scale bar = 100 *μ*m).

Our previous publications demonstrated that CHS could significantly reduce the innate immune response and then increase the susceptibility of animals to H5N1 virus [2]. To determine if this effect holds true for vaccinations against H5N1, we vaccinated mice in neutrally thermal or chronic heat conditions. As outlined in Figure 1, mice were divided into four groups. Mice were either subjected to chronic heat stress and immunized with inactivated virus (CHS+Ag) or a PBS control (CHS+PBS) or kept in a thermally neutral environment and immunized with inactivated virus (TN+Ag) or a PBS control (TN+PBS). Seven days after the second vaccination (day 21) mice were challenged with the H5N1 virus, and the mortality was observed for 14 days. As shown in Figure 2(a), mortality rates of the CHS+Ag group and the TN+Ag group were 16.7% and 0%, respectively. These rates were lower than those of the CHS+PBS (66.7%, *P* = 0.0563) and TN+PBS groups (50%, *P* = 0.0549), suggesting that the vaccination strategy was protective. However, the mortality rate of CHS+Ag group was higher than the one of TN+Ag group (*P* = 0.1673), suggesting that CHS does negatively impact the immune response to vaccination. To examine the possible inhibition of CHS on the immune efficacy, viral loads in lungs of mice on day 3 and day 6 postinfection were determined by real-time PCR. As shown in Figure 2(b), the viral load in the CHS+Ag group was higher than it was in the TN+Ag group on day 3 and day 6 postinfection (*P* < 0.05). To determine the effect of this heightened viral load, histopathological changes in the lungs of mice in each group at day 6 postinfection were examined (Figure 2(c)). As expected the lung of the control groups displayed severe lesions including interstitial pneumonia with interstitial oedema and inflammatory cellular infiltration around small blood vessels, dropout of the mucous epithelium adhering to the surface of bronchioles, thickening of alveolar walls, and alveolar lumen flooded with oedema fluid mixed with exfoliated alveolar epithelial cells, erythrocytes, and inflammatory cells. The degree of histopathologic changes displayed correlated with the level of the viral load in that mice in the CHS+PBS group displayed more severe pathological changes than mice in the TN+PBS group. In contrast, the lungs of mice in the TN+Ag group showed no apparent histological changes. However, mice in the CHS+Ag group displayed blood capillary congestion, mild oedema, and a small quantity of inflammatory cell infiltration near the small blood vessels. Therefore, these data suggest that vaccination with formalin-inactivated virus can stimulate a protective immune reaction against the H5N1 virus infection; however, the protective efficacy is significantly inhibited by CHS. 

### 3.2. CHS Negatively Influences the Immune Effects Induced by Formalin-Inactivated H5N1 Virus

**Figure 3:**
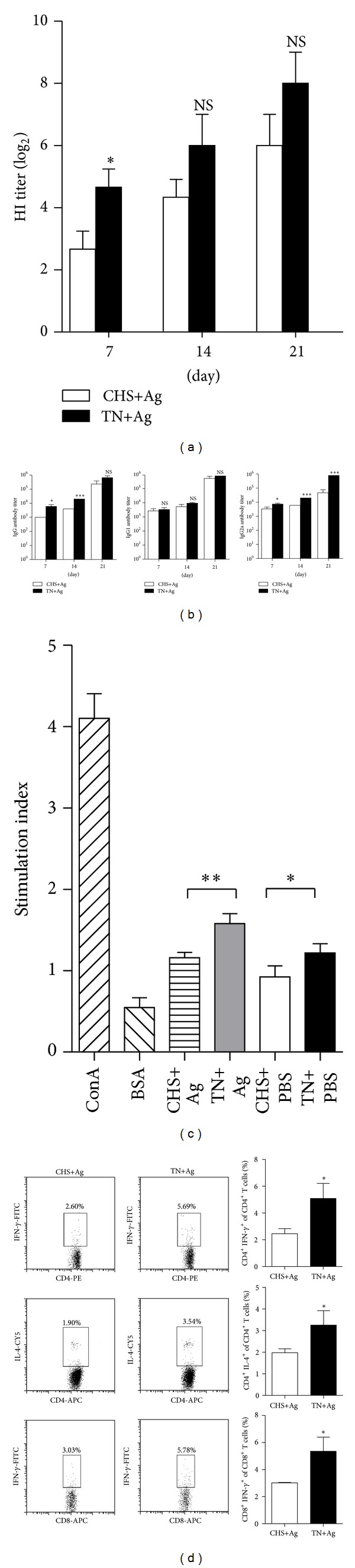
CHS negatively influences the immune effects induced by formalin-inactivated H5N1 virus. Serum samples and T cells of three mice from CHS+Ag and TN+Ag groups were collected on indicated days postprimary immunization. (a) Antibody levels against the H5 subtype antigen were expressed by HI titer. (b) Ag-specific IgG, IgG1, and IgG2a were determined by ELISA. (c) T cells were isolated on day 21 and stimulated with specific antigen; cell proliferation was determined by MTS/PMS method. (d) T cells collected on day 21 were stimulated with a viral-specific antigen for 6 h *in vitro*, stained with different florescent antibodies, and analyzed by flow cytometry. The numbers in the box indicate the percentages of examined cytokines. Data are summarized in a bar graph to the right. Results shown are pooled from three independent experiments.

The increased mortality in CHS treated groups could be due to the inhibition of protective immunity. To examine whether CHS can influence the immune function of vaccination, HI tests were conducted to detect the levels of H5 subtype antibodies in serum samples. As shown in Figure 3(a), HI titers ascended in accordance with the immunization process in both the CHS and TN groups; however, titers of the CHS+Ag group were lower than TN+Ag group at all time points, especially on day 7 (*P* < 0.05). Similarly, IgG and IgG2a titers, as determined by ELISA, in the CHS+Ag group were significantly lower than TN+Ag group at multiple time points (Figure 3(b)), but IgG1 titers were not significantly different between the two groups. These data suggest that CHS can negatively affect virus-related antibody production and influence the humoral response in the host.

To test the effect of CHS on T-cell responses, single-cell suspensions were isolated from spleens of the immunized mice on day 7 after the last immunization. As shown in Figure 3(c), the antigen specific T-cell proliferative response was significantly inhibited by CHS (*P* < 0.01), so did the nonspecific proliferation (*P* < 0.05). The effect of CHS on T-cell cytokine production was also examined by flow cytometry. The percentage of antigen induced IFN-*γ* and IL-4 in CD4^+^ T cells was significantly suppressed after CHS (Figure 3(d); *P* < 0.05). These results suggest that CHS can suppress the productions of both Th1 and Th2 cytokines. In addition, a lower percentage of CD8^+^ T cells expressing intracellular IFN-*γ* was observed in mice subjected to CHS (*P* < 0.05), indicating that the CHS treatment may suppress antigen specific CTL response. Collectively, these results support the idea that CHS can negatively affect host humoral and cell-mediated immunity induced by vaccination.

### 3.3. CHS Regulates Tregs Prevalence

**Figure 4:**
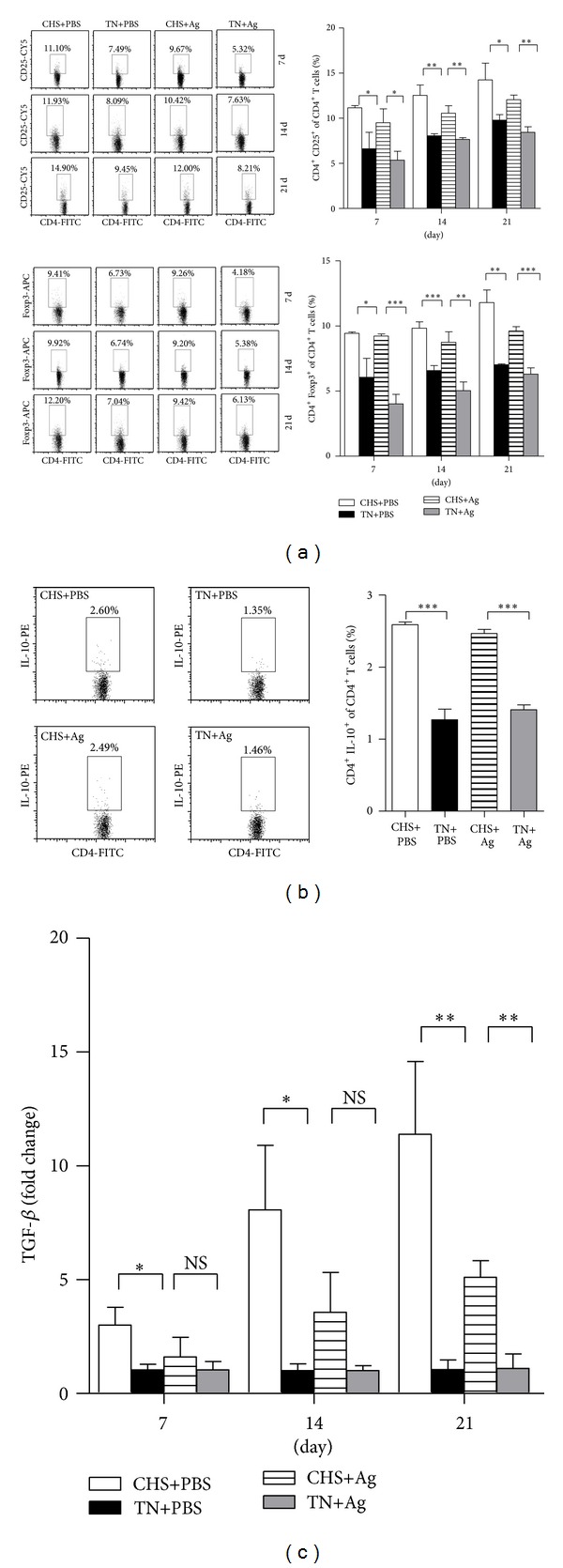
CHS regulates Tregs prevalence. Splenic T cells from each group (*n* = 3) were isolated on the indicated days postprimary immunization and then analyzed by flow cytometry. (a) Percentages of CD25^+^ and Foxp3^+^ cells in CD4^+^ T cells were calculated at the indicated time points. Results shown are pooled from three independent repeats. (b) Intracellular staining of IL-10^+^ CD4^+^ T cells on day 21 was performed (stimulated with a viral-specific antigen for 6 h *in vitro* before being analyzed by flow cytometry). Representative plots showing the percentages of positive T cells are shown on the left. Data from all experiments are summarized in bar graphs to the right. (c) The expression levels of TGF-*β* in the CD4^+^ cells of CHS groups were analyzed by real-time PCR at indicated time points. Data are presented as relative changes in gene expression (fold) normalized to the TN groups and are one representative from three independent experiments.

Previous reports support a role for regulatory T cells (Tregs) in suppressing immunity [17]. Given the reduced immune response observed when mice were subjected to CHS, we examined whether CHS also altered the expression of Tregs. To this end we analyzed the percentages of CD4^+^CD25^+^ and CD4^+^Foxp3^+^ cells within the total CD4^+^ T-cell population by flow cytometry. Over the course of 21 days, the percentages of CD4^+^ expressing CD25^+^ T and the transcription factor Foxp3 (a unique hallmark of Tregs) were elevated in the CHS compared to the corresponding TN groups (Figure 4(a)). Together these data suggest that CHS increases the percentage of Tregs within the CD4^+^ population. One way Tregs suppress the immune response is by secreting suppressive cytokines. Thus, the expression levels of Tregs-related cytokines were also evaluated. The expression of the anti-inflammatory cytokine IL-10 in the CHS groups on day 7 after the boost immunization was significantly elevated compared to the control groups (Figure 4(b): *P* < 0.001). Consistent with IL-10 expression, the fold change of TGF-*β* levels in the CHS groups were also increased (Figure 4(c)). We also detected the expression levels of IL-35, but there were no differences between the CHS and control groups (data not shown). Taken together, these data suggest that CHS alters the prevalence of Tregs and the secretion of anti-inflammatory cytokines, IL-10 and TGF-*β*.

### 3.4. Adoptive Transfer of CD4^+^CD25^+^ Tregs Reduces the Vaccine Efficacy under TN Conditions

**Figure 5:**
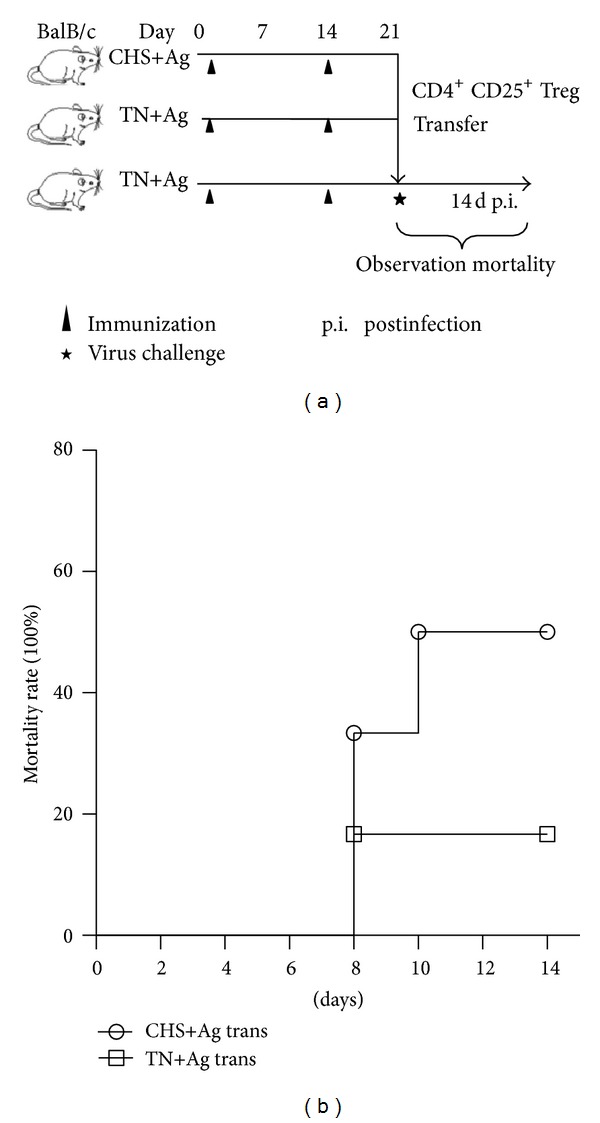
Adoptive transfer of CD4^+^CD25^+^ Tregs reduces the vaccine efficacy under TN conditions. 10^6^ CD4^+^CD25^+^ Tregs from CHS+Ag group and TN+Ag group were adoptively transferred into TN+Ag mice on day 21 in conjunction with a 50 PFU H5N1 virus challenge. The mortalities of the two transferred groups (*n* = 6) were observed for 14 days.

Because CHS increased the percentage of Tregs, we hypothesized that CHS-induced Tregs might suppress the protective efficacy of vaccination under TN conditions. To test this, we adoptively transferred CD4^+^CD25^+^ Tregs (including CD4^+^CD25^+^Foxp3^−^ T cells because of the limited laboratory techniques) from either the CHS+Ag group or the TN+Ag group into TN+Ag mice in conjunction with the H5N1 virus challenge and observed mice mortality (Figure 5(a)). The mortality of mice that received Tregs from the CHS+Ag group was 50.0%, whereas mice that received Tregs from the TN+Ag group was only 16.7% (Figure 5(b); *P* = 0.0852). In addition, the mortalities of both groups receiving Tregs were elevated compared with the normal CHS+Ag group (16.7%) and TN+Ag group (0%) (Figure 2(a)). In order to examine the effects of non-Treg CD25^+^ cells used in adoptive transfer experiments, we detected the percentages of CD4^+^Foxp3^−^ T-cell population in each group, and there was no statistic differences between CHS+Ag and TN+Ag group (data not shown). The results indicate that the existence of non-Treg CD25^+^ cells in adoptive transfer experiments cannot impact the results significantly. The results previously demonstrate that Tregs induced by CHS weaken the protective efficacy of formalin-inactivated virus immunization and that this suppression can be transferred.

## 4. Discussion

Regulatory T cells (Tregs) play a critical role in suppressing the host immune response [17, 18]. Past studies have demonstrated that chronic heat stress (CHS) can have negative impacts on the host response to vaccination, but the role of Tregs in this process remains largely underexplored. In this study we demonstrate that CHS regulates Tregs, which inhibited the protective efficacy induced by vaccination to the H5N1 virus *in vivo*. Supporting this observation, CHS (1) increased the frequency of CD4^+^CD25^+^ Foxp3^+^ Tregs, (2) heightened levels of IL-10 and TGF-*β* from CD4^+^ cells, (3) suppressed Th1 and Th2 immune responses to reduce antibody titers, and (4) reduced CD8^+^ T-cell proliferation and related cytokines.

It is well known that the protective immunity against viral infections is correlated with both antigen-specific humoral and cell-mediated responses [19, 20]. Previously, we demonstrated the negative effects of CHS on the immune efficacy of DNA vaccination [1]. These previous results suggested that the cell-mediated responses induced by FMD DNA vaccination were suppressed significantly, particularly to the antigen-specific CTL activities *in vivo*; however, the humoral responses, as measured by total IgG and IgG1, were only slightly affected CHS. Consistent with these previous observations, the data presented here suggest that CHS can reduce the cellular immune response induced by formalin-inactivated H5N1 virus immunization. Unlike our previous results, however, CHS also significantly suppressed the humoral response. To further understand if the reduced levels of the humoral and cell mediated responses induced by CHS correlated with the protective efficacy of inactivated virus immunization, the mortality rates, viral loads, and lung lesions were observed after H5N1 virus challenge. Indeed, CHS significantly increased mortality rates, viral loads, and lung pathology even in vaccinated mice. Thus, CHS weakened the protective efficacy by suppressing both the cell mediated and humoral responses of formalin-inactivated H5N1 virus immunization. 

Two main types of regulatory T cells have been identified, naturally (nTreg) and inducible (iTreg) a regulatory T cells, which were important mediators of immune homeostasis with naturally endowed immune-suppressive activity [21–23]. To date, multiple Tregs subsets (e.g., nTreg, cells, Th3 cells, CD8 Treg, etc.) have been identified which can exert negative immunoregulatory effects on the activation and effector functions of innate and adaptive immune cells by different mechanisms [21]. It was reported that Tregs played a major role in regulating immune responses to vaccination. Most Tregs were reported to date have been CD25^+^ cells (CD4^+^CD25^+^Foxp3^+^), and it is well established that their induction requires suboptimal stimulation of the T-cell receptor (TCR) and related cytokines [8]. In the present study, we demonstrated that CHS induced the presence of CD4^+^CD25^+^Foxp3^+^ Tregs, and this correlated with the downregulation of both Th1 and Th2 immune response stimulated by H5N1 influenza vaccination in mice. In addition, the levels of IL-10 and TGF-*β* from CD4^+^ cells were increased; the secretions of these suppressive cytokines are one way that Tregs suppress the immune response, such we speculate that the increased secretions of IL-10 and TGF-*β* in CD4^+^ T cells might be produced by CD4^+^CD25^+^Foxp3^+^ Tregs, although further experiments need to confirm these. Consistent with this observation, antigen-specific and nonspecific T-cell proliferative responses were significantly reduced when mice were exposed to CHS. Moreover, CHS dramatically decreased the level of IFN-*γ* both in CD4^+^ and CD8^+^ T cells, which is a signature Th1 cytokine responsible for protection against a variety of intracellular infections [24]. Furthermore, adoptive transfer of the Tregs from CHS mice was able to suppress the protective efficacy of formalin-inactivated H5N1 virus immunization in TN mice. These results suggest that CHS-induced CD4^+^CD25^+^Foxp3^+^ Tregs weaken the protective efficacy against H5N1 virus infection in mice. However, the mechanisms for Treg induction under CHS were still unknown. We speculated that the Treg cells induction under CHS condition might include both nTreg and iTreg. CHS itself gradually increases TGF-*β* as the duration of CHS becomes longer (Figure 4(c)). It could be possible that increased TGF-*β* during CHS induces iTreg, which need to be further verified.

## 5. Conclusions

In summary, this study exposes a novel mechanism of immunosuppression following CHS whereby increases the number of CD4^+^CD25^+^Foxp3^+^ Tregs and the level of IL-10 and TGF-*β* and suppresses adaptive immune responses stimulated by H5N1 influenza vaccination in mice. These data clarify the mechanism of the decreased protective efficacy of formalin-inactivated H5N1 virus immunization in mice under CHS conditions and open a new venue for treating immunosuppression caused by CHS.
